# The Role of Autophagy Dysregulation in Manganese-Induced Dopaminergic Neurodegeneration

**DOI:** 10.1007/s12640-013-9392-5

**Published:** 2013-04-19

**Authors:** Jianbin Zhang, Rui Cao, Tongjian Cai, Michael Aschner, Fang Zhao, Ting Yao, Yaoming Chen, Zipeng Cao, Wenjing Luo, Jingyuan Chen

**Affiliations:** 1 Department of Occupational & Environmental Health and the Ministry of Education Key Lab of Hazard Assessment and Control in Special Operational Environment, School of Public Health, Fourth Military Medical University, 169 Changlexi Road, Xi’an, 710032 China; 2 Department of Pediatrics, Vanderbilt University Medical Center, Nashville, TN USA

**Keywords:** Manganese, Autophagy, Dopaminergic neuron, Beclin 1

## Abstract

The etiological role of dysregulated autophagy in neurodegenerative diseases has been a subject of intense investigation. While manganese (Mn) is known to cause dopaminergic (DAergic) neurodegeneration, it has yet to be determined whether the dysregulation of autophagy plays a role in Mn-induced neuronal injury. In this study, we investigated the effect of Mn on autophagy in a rat model of manganism, a neurodegenerative disease associated with excessive exposure to Mn. After a single intrastriatal injection of Mn, the short- (4–12 h) and long-term (1–28 days) effect of Mn on DAergic neurons and autophagy were examined. Marked reduction in the number of TH-immunoreactive neurons in the substantia nigra pars compacta (SNpc) as well as TH protein expression, and a significant increase of apomorphine-induced rotations were observed in rats after Mn injection. Manganese also induced the down-regulation of dopamine levels and D_1_ dopamine receptor expression. In addition, autophagy was dysregulated and inhibited, as evidenced by increased number of abnormal lysosomes, decreased protein expression of Beclin1, and decreased ratio of microtubule-associated protein 1 light chain 3 (LC3) II over LC3 I, concomitant with activated mammalian target of rapamycin (mTOR)/p70 ribosomal protein S6 kinase (p70s6k) signaling. In contrast, in the early phase of Mn exposure, the level of autophagy was not be suppressed but compensatorily activated. Although the morphology of the DAergic neuron was intact in the early phase, Mn caused a significant decrease in TH-immunoreactivity and a significant increase in apomorphine-induced rotations in the presence of wortmannin, an inhibitor of autophagy. Taken together, these results demonstrate, for the first time, that autophagy may play a protective role against Mn-induced neuronal damage, whilst dysregulation of autophagy at later phases may mediate DAergic neurodegeneration.

## Introduction

As one of the most abundant element in the earth’s crust, Mn is an essential element for humans, animals, and plants, and is required for growth, development, and maintenance of health. Mn is necessary for a variety of metabolic functions, including lipid, protein, and carbohydrate metabolism, and it serves as a cofactor for various enzymes, including the antioxidant enzyme superoxide dismutase (SOD), as well as enzymes involved in neurotransmitter synthesis and metabolism (Carl et al. 1993; Keen et al. 2000; Johnson and Giulivi 2005; Aschner et al. 2007). However, exposure to high levels of Mn may lead to a neurological disorder that shares many similarities with Parkinson’s disease (PD), and is referred to as manganism. Within the central nervous system (CNS), exposure to high levels of Mn in occupational or environmental settings or disease conditions is accompanied by Mn accumulation in specific brain regions that are highly sensitive to oxidative injury, including the global pallidus (GP), striatum (STR) and substantia nigra (SN) (Newland et al. 1989; McKinney et al. 2004; Benedetto et al. 2010). DAergic neurodegeneration in the SN has been reported in manganism (Levy and Nassetta 2003). The disorder was firstly reported by John Couper in 1837 in five men working in a Mn ore crushing plant in France, characterized by muscle weakness, limb tremor, whispering speech, salivation, and a bent posture. The most frequent cause of Mn neurotoxicity is believed to be chronic occupational exposure to high levels of inhalable manganese (>1–5 mg Mn/m^3^), which is commonly associated with occupations, such as Mn mining and smelting, battery manufacturing, and steel production (Santamaria et al. 2007) .

Autophagy is a general term referring to pathways by which cytoplasmic materials, including soluble macromolecules and organelles, are delivered to lysosomes for degradation, thus eliminating defective cell structures (Mizushima et al. 2010). Accordingly, imbalance in autophagy has been implicated in several diseased states. Recent studies reveal the degradation of disease-related mutant proteins is highly dependent on autophagy, in addition to the ubiquitin–proteasome system (Li and Li 2011). Examples include extended polyglutamine-containing proteins that cause various neurodegenerative diseases, such as Huntington’s disease and spinocerebellar ataxia, and mutant forms of α-synuclein that causes familial PD (Zhao et al. 2009). Dysregulation of autophagy has been linked to several neurodegenerative diseases (Jung et al. 2010; Alirezaei et al. 2011, Chen and Klionsky 2011; Fan and Weiss 2011). In contrast, up-regulation of autophagy has been found to afford neuroprotection, delaying, or ameliorating these disorders (Alirezaei et al. 2011). However, the role of dysregulated autophagy in mediating Mn-induced neurotoxicity has yet to be addressed. In the present study, we investigated the effect of Mn on autophagy and the role of its dysregulation in Mn-induced DAergic neuronal damage.

## Materials and Methods

### Materials and Reagents

Polyclonal anti-tyrosine hydroxylase antibody (TH) was obtained from Chemicon (Temecula, CA, USA). Neu-N antibody was purchased from Santa Cruz Biotechnology (Santa Cruz, CA, USA). Other chemicals and reagents were purchased from Cell Signaling Technology (Inc., Danvers, MA, USA).

### Animals and Treatment

All procedures involving animals were carried out in strict accordance with the international standards of animal care guidelines and were approved by the institutional animal care and use committee of Fourth Military Medical University (Permit Number:12002). Male Sprague–Dawley rats (180–210 g) were purchased from the Experimental Animal Center of Fourth Military Medical University. They were maintained in a standard environmental condition and fed with a standard pellet diet and water ad libitum. The rats were housed in stainless steel cages in a room kept at 22 ± 1 °C with a 12-h light/12-h dark cycle.

Rats were injected with Mn as we previously described (Zhao et al. 2009). In brief, rats were anaesthetized with sodium pentobarbital (60 mg/kg ip) and placed in a stereotaxic frame with the nose bar set at −2.4 mm. For manganese intoxication, rats were stereotaxically injected in the right striatum with 1 μl MnCl_2_·4H_2_O (1 mol/L) or 0.9 % NaCl. The following stereotaxic co-ordinates were used: 1 mm anterior-posterior, 3 mm lateral, 4.5 mm ventral to the dura. The injections were slowly infused over 1 min to avoid tissue damage and the needle remained in situ for additional 8 min prior to its withdrawal. Wortmannin (100 mg/kg) treated rats were i.p. injected 30 min prior to the Mn or saline injection. For long-term treatments, observations, and analyses were conducted at 1, 7, 14, and 28 days postinjections. For short-term treatments, observations, and analyses were conducted 4, 8, and 12 h post injection. At the end of the experiments, rats were sacrificed and the brains were collected for morphological and biochemical analyses.

### Apomorphine-Induced Rotations

Five minutes after the i.p. injection of 0.6 mg/kg apomorphine, each rat was placed in a chamber (1 × 1 m^2^) and the rotational asymmetry (ipsilateral–contralateral) score was recorded for 30 min following the injection, as previously described (Mandel 2000).

### Histological Observation

Immediately upon removal, the brains were fixed in 10 % buffered formaldehyde, and processed for histological examination according to conventional methods, followed by hematoxylin and eosin (H&E) staining.

### Immunostaining

Immunostaining was carried out as we previously reported (Zhao et al. 2009). In brief, rats were cordially perfused under deep anesthesia with 150–200 ml of 4 % paraformaldehyde in phosphate buffer, pH 7.4. Then brains were removed, and cryoprotection was carried out in 25 % sucrose phosphate buffer, pH 7.4. Next, the brains were frozen at −20 °C, and serial sections (10 μm) were cut and mounted on glass slides. Nonspecific antibody-binding sites were blocked with 1 % bovine serum albumin (BSA; Sigma-Aldrich, St Louis, MO) in PBS, after which slides were incubated with primary antibodies. The anti-TH antibody was used to identify DAergic neurons by conventional immunocytochemistry techniques. Neurons were identified by immunocytochemistry with an anti Neu-N antibody. The sections were visualized using a mixture of fluorescein isothiocyanate (FITC)-conjugated anti-mouse (diluted 1:400; Sigma) and Cy3-conjugated anti-rabbit (diluted 1:600; Sigma) antibodies. Sections were examined with a FV300 laser scanning microscope (Olympus, Tokyo, Japan).

The total number of TH-positive DAergic neurons and Neu-N-positive neurons in substantia nigra pars compacta (SNpc) were counted in various groups of animals at 8 h, 1, 7, 14, and 28 days after the treatment using the following method as described previously (Iravani et al. 2005). The number of tyrosine hydroxylase-immunoreactive (TH-ir) neurons at the level of the third nerve was obtained by manually counting the total TH-ir neurons from three to seven adjacent sections, and divided by the number of sections. Based on the counting of DAergic neurons throughout the SNpc at regular 100-μm intervals, it was previously shown that the third nerve rootlets provide a reliable anatomical landmark at which the extent of cell loss can be accurately assessed and the extent of cell loss at this point is reflective of cell loss throughout the entire structure (Iravani et al. 2002). The extent of dopamine neuronal loss was estimated by counting the number of TH-ir SNpc neurons at the level of the third nerve rootlets on the lesioned side compared with that of control rats. All cells that appeared severely deformed were excluded from the count.

### Transmission Electron Microscopy (TEM)

TEM was conducted as previously reported (Ye et al. 2010). In brief, at the end of the experiments, brains were collected and fixed in 2 % glutaraldehyde in 0.1 M PBS (pH 7.4) for 2 h at 4 °C. Subsequently, they were post-fixed in 1 % osmium tetroxide in 0.1 M PBS (pH 7.4) for 2 h at 4 °C. Next, the SN tissue was dissected out (1 mm^3^) and sequentially processed in 1 % osmium tetroxide, dehydrated in ascending ethanol solutions ranging from 20 to 100 %, and embedded in E-pon812. Ultrathin sections (80 nm) were impregnated in 2 % uranyl acetate and Reynold’s lead citrate. Sections were viewed with transmission electron microscope (JEM-2000EX, Japan).

### Western Blotting

Western blotting was conducted as previously described (Wang et al. 2011). In brief, brains were incubated on ice with lysis buffer and centrifuged at 20,000×*g*. The supernatant was collected and protein concentrations were determined with the Pierce Bicinchoninic acid (BCA) Protein Assay Kit (Thermo). Next, the supernatant was mixed with equal volume of sample buffer and the mixture was boiled for 5 min and centrifuged for 10 min at 10,000×*g*. Proteins were separated by SDS-PAGE on 5–12 % polyacrylamide gels, and transferred to nitrocellulose membranes (Millipore, Billerica, MA, USA). After blocking for 1 h with 5 % skimmed milk in TBS buffer (10 mM Tris, 150 mM NaCl), the membranes were probed with primary antibodies, including rabbit anti-Human TH polyclonal antibody (1:1,000 dilution, Chemicon, Temecula, CA, USA), mouse anti-β-actin monoclonal antibody (1:1,000 dilution, Santa Cruz Biotechnology, CA, USA), rabbit anti-Beclin 1 polyclonal antibody (1:1,000, Cell Signaling Technology, Inc., Danvers, MA, USA), anti-D_1_ dopamine receptor polyclonal antibody (1:1,000 dilution, Sigma-aldrich, USA**)**, rabbit anti-LC3 antibody (1:500, Cell Signaling Technology, Inc., Danvers, MA, USA), rabbit anti-p70S6K antibody (1:1,000, Cell Signaling Technology, Inc., Danvers, MA, USA), rabbit anti-phospho p70S6K antibody (1:1,000, Cell Signaling Technology, Inc., Danvers, MA, USA), rabbit anti-mTOR antibody (1:1,000, Cell Signaling Technology, Inc., Danvers, MA, USA), rabbit anti-phospho mTOR antibody (1:1,000, Cell Signaling Technology, Inc., Danvers, MA, USA) at 4 °C overnight. Next, the membranes were washed four times, 15 min each, with TBST buffer (10 mM Tris, 150 mM NaCl, and 0.1 % Tween-20) and incubated for 30 min at 37 °C with appropriate HRP-conjugated secondary antibodies. The protein bands were visualized with chemiluminescent reagents following the manufacturer’s instructions, and exposed to Hyperfilm-ECL (Amersham). Densitometry analysis of band intensity was performed using Image J software.

### Enzyme-Linked Immunoassay (ELISA)

Rats were sacrificed and brain tissues were quickly removed, frozen in liquid nitrogen and stored at −80 °C for posterior biochemical analysis. The levels of DA were measured by ELISA according to the manufacturer’s (Xi Tang, Biotech, Shanghai, China) instructions. A 50 mg portion of the homogenate was diluted in 500 μl normal saline (0.9 %) for detection. The DA in supernatant fluid was assayed by using the ELISA kit (Xi Tang, Biotech, Shanghai, China) after centrifugation of homogenized tissue for 10 min at 14,000×*g* at 4 °C. Dispensed antigen standards and samples were added to each well of 96-well plates precoated with primary antibodies. After adding biotin conjugate reagent and enzyme conjugate reagent into each well, the plates were incubated at 37 °C for 30 min. Then the plates were rinsed five times with distilled water. Within 30 min of the chromogenic reaction, the absorbance was measured at 450 nm using a microtiter plate reader (TECAN, Swiss).

### Statistical Analysis

Results were expressed as the mean ± SD and were analyzed by one-way analysis of variance (ANOVA) followed by a SNK-*q* test for multiple comparisons. Student *t* test was used for two group comparisons. All analyses were performed with SPSS 16.0 software. Data were considered statistically significant at *p* < 0.05.

## Results

### Effect of Mn on Apomorphine-Induced Rotation

**Fig. 1:**
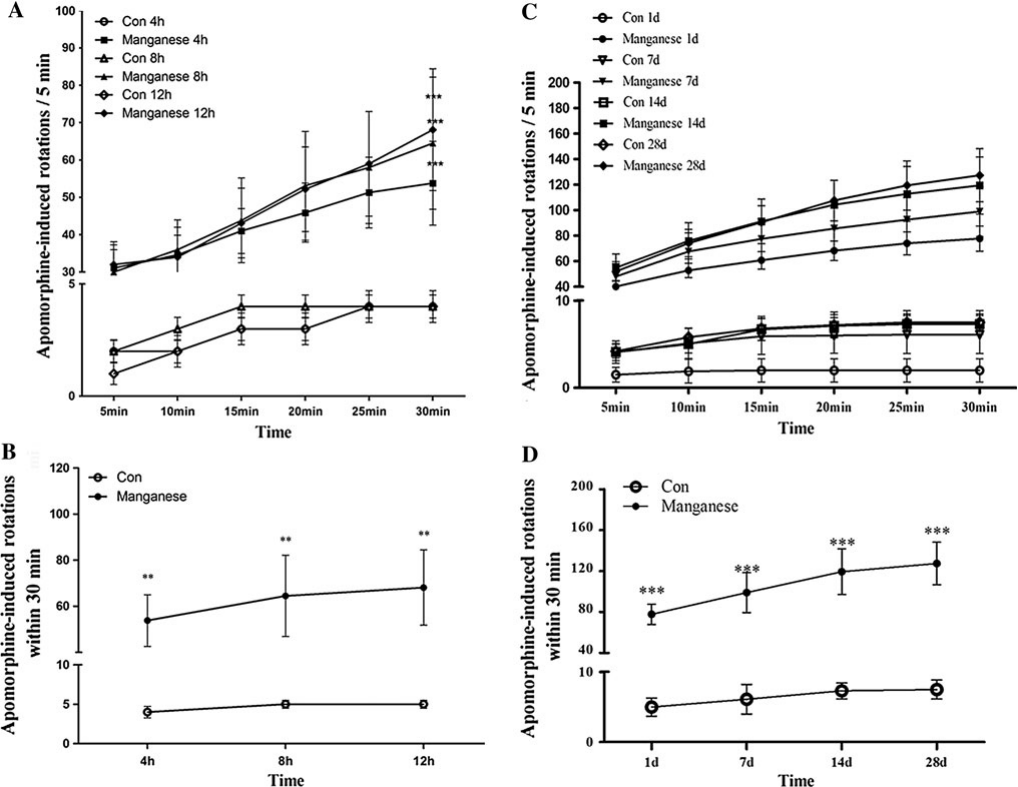
The effect of manganese on apomorphine-induced rotations. Different time points after manganese administration, rats were dosed with i.p. injection of 0.6 mg/kg apomorphine, and then placed in a chamber (1 × 1 m^2^) in a testing room and their rotational asymmetry (ipsilateral–contralateral) score was monitored for 30 min (*n* = 10). **a**, **c** Rotational behavior induced by apomorphine was assessed after short-term (4, 8, and 12 h) and long-term (1, 7, 14, and 28 days) periods. The number of full 360° rotations in the *ipsilateral* direction was counted for 30 min after the administration of apomorphine. Significance (Bonferroni/Dunn post hoc comparisons after ANOVA): *p < 0.05, **p < 0.01 versus the vehicle control at each time point. **b**, **d** Time-course of changes in the number of apomorphine-induced ipsilateral rotations at different time points in rats injected with vehicle control, or Mn, or wortmannin. Graphs show mean ± SD. *p < 0.05, **p < 0.01, compared with respective control. Student *t* test was used for two group comparisons

Previous studies have shown that if the DAergic neurons are damaged in SNc, there will be abnormal behaviors. Apomorphine-induced abnormal rotational behavior after Mn injection was evaluated as a surrogate of DAergic neuronal integrity (Ungerstedt 1971). In order to detect the damage after Mn exposure, we observed apomorphine-induced rotational behavior. As shown in Fig. 1, apomorphine-induced rotations were significantly increased after that rats were exposed to Mn for short-term [4, 8, and 12 h post injection (Fig. 1a, b)] and long-term [1, 7, 14, and 28 days post injection (Fig. 1c, d)] periods.

### Effect of Mn on Substantia Nigral DAergic Neurons

**Fig. 2:**
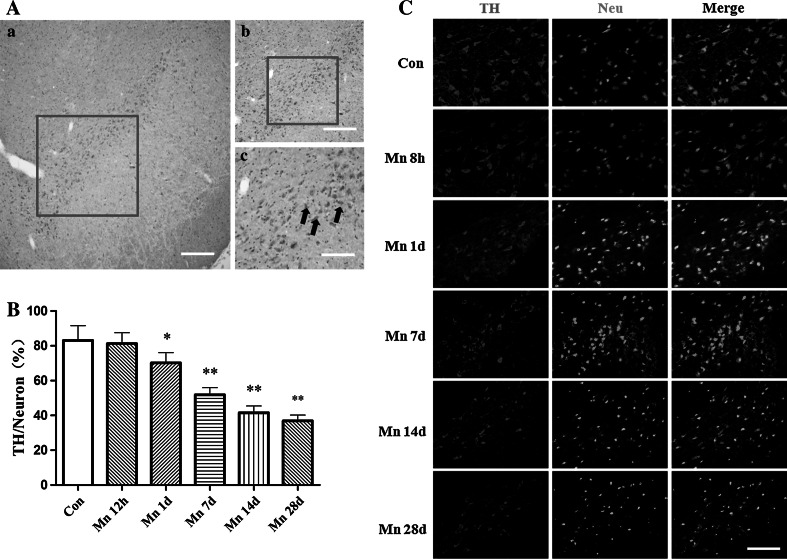
The effect of manganese on DAergic neurons in immunofluorescence. 8 h, 1, 7, 14, and 28 days after manganese administration, immunofluorescence was conducted to measure TH-immunoreactivity and TH expression. **A** Location of substantia nigra pars compacta (SNpc). **a** *Scale bar* indicates 1,000 μm; **b** *Scale bar* indicates 500 μm; **c** *Scale bar* indicates 200 μm. **B** Quantitative analysis of the effect of manganese on DAergic neurons in the ratio of total neurons. *p < 0.05, **p < 0.01 compared with control groups. **C** The effect of manganese on TH-immunoreactivity. *Scale bar* indicates 200 μm

**Fig. 3:**
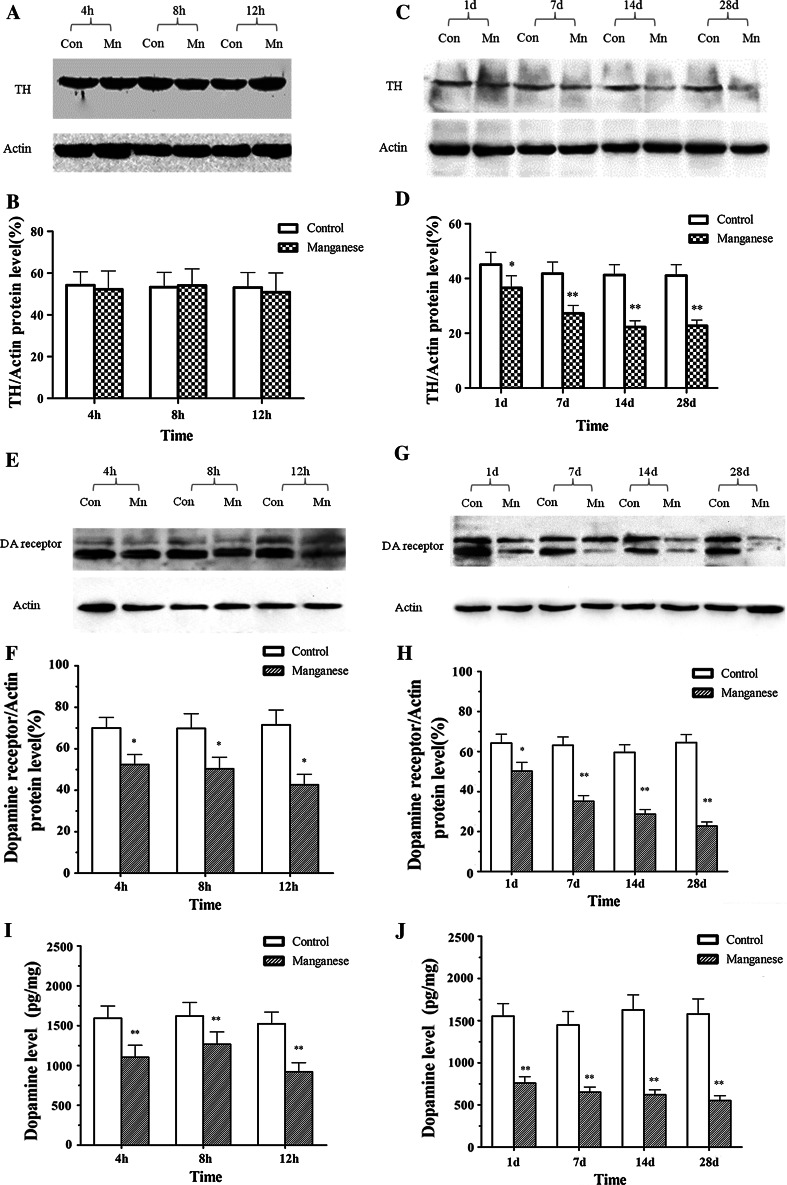
The effect of manganese on DAergic neurons in Western blotting. Short- (4, 8, and 12 h) and long-term (1, 7, 14, and 28 days) after manganese administration, Western blotting was conducted to measure TH expression. **a**, **c** Effect of manganese on TH protein expression. **b**, **d** Densitometry analysis of TH levels relative to β-actin was performed after three independent experiments. **e**, **g** Effect of manganese on D_1_ dopamine receptor protein expression. **i**, **j** Effect of manganese on dopamine levels. **f**, **h** Densitometry analysis of D_1_ dopamine receptor levels relative to β-actin was performed using three independent experiments (mean ± SD; one-way ANOVA with Newman–Keuls post hoc analysis, *p < 0.05 and **p < 0.01)

Histological evaluation was conducted to observe the changes in the substantia nigra pars compacta (Fig. 2A). Immunostaining was performed with antibodies against TH and Neu-N. As shown in Fig. 2B, no obvious change of TH-immunoreactivity was observed at 8 h post intrastriatal Mn injection. However, from 1 day after intrastriatal Mn injection, there were significant difference in TH-immunoreactivity compared with that of control group (Fig. 2C). Consistent with this effect, the protein expression of TH unaltered 4, 8 and 12 h post-Mn injection (Fig. 3a), but 1 day after Mn injection, TH protein levels in the SNc were decreased mildly, and 7–28 days after Mn injection, TH protein levels were decreased significantly, compared with that of control group (Fig. 3c, d).

The content of dopamine was determined by ELISA. As shown in Fig. 3i, as early as 4 h after intrastriatal Mn injection, there was significant difference in dopamine levels compared with controls. Moreover, the dopamine receptor protein level in the SNc decreased mildly 4, 8, 12 h after Mn injection, but significantly 1, 7, 14, 28 days after Mn injection, compared with that of control group (Fig. 3e, g).

### Effect of Manganese on Autophagy

**Fig. 4:**
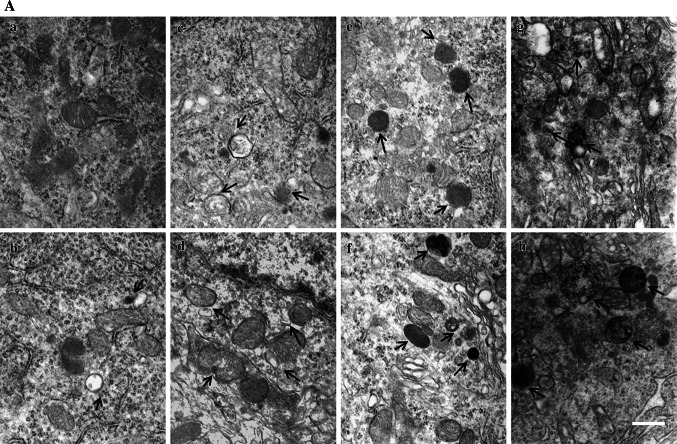

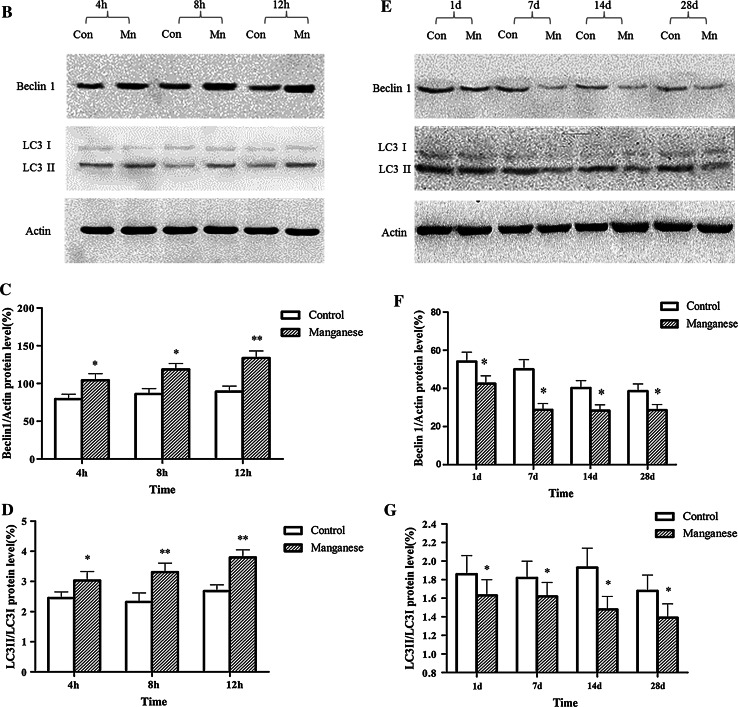
The effect of Mn on autophagy. Short- (4, 8, and 12 h) and long-term (1, 7, 14, and 28 days) after Mn injection, TEM and Western blotting were conducted to evaluate autophagy. **a** TEM observation on increased autophagy (**a** control; **b**, **c**, **d** 4, 8, and 12 h post Mn injection) and dysfunctional lysosomes (**e**–**h** 1, 7, 14, and 28 days post Mn injection). Figure 4 **A**, **b**–**d** shows that the autophagosomes-containing double membrane increased compared with that of control, Fig. 4 **A**, **e**–**h** shows that increased dysfunctional lysosomes-containing dense granules compared with the control. **b**, **e** Effect of Mn on protein expression of Beclin 1, LC3 II, and LC3 I. **c**, **d**, **f**, **g** Densitometry analysis of Beclin 1 protein levels and LC3 II levels relative to LC3 I was performed using three independent experiments. β-Actin was used as control for protein loading. Above results were obtained after three independent experiments (mean ± SD; one-way ANOVA with Newman–Keuls post hoc analysis, *p < 0.05 and **p < 0.01)

To evaluate the effect of Mn on autophagy, TEM and Western blotting studies were carried out. As shown in Fig. 4A, the number of autophagosome (the product of fusion of autophagic vacuole lysosome) in Mn-injected rats notably increased, concomitantly with slight swollen mitochondria 4 (Fig. 4A–B), 8 (Fig. 4A–C), 12 h(Fig. 4A–D) after the administration. However, TEM showed progressive effect of intrastriatal Mn injection on several morphometric parameters (Fig. 4A, e–h), characterized by increased number of mitochondrial vacuolus, swollen, and broken endoplasmic reticulum, and dysfunctional lysosomes containing high dense particulate materials and decreased autophagic vacuoles in DAergic neurons in the SNpc of Mn-exposed rats. These changes suggest the attenuated rate of autophagy, which is consistent with a former report (Kruger et al. 2011). Additional western blotting experiments established two-phase effect of Mn on Beclin-1 and microtubule-associated protein 1 light chain 3 (LC3) protein expression in different phases. As shown in Fig. 4, Beclin 1 protein expression and the ratio of LC3 II to LC3 I were significantly increased by Mn after short-time periods (Fig. 4B), but significantly and time-dependent (1, 7, 14, and 28 days) decreased after long time periods (Fig. 4e). In a word, manganese-induced compensatory autophagy activation in short terms and inhibited autophagy in long terms.

### Effect of Mn on Autophagy-Related Signaling Pathway mTOR/p70S6K

**Fig. 5:**
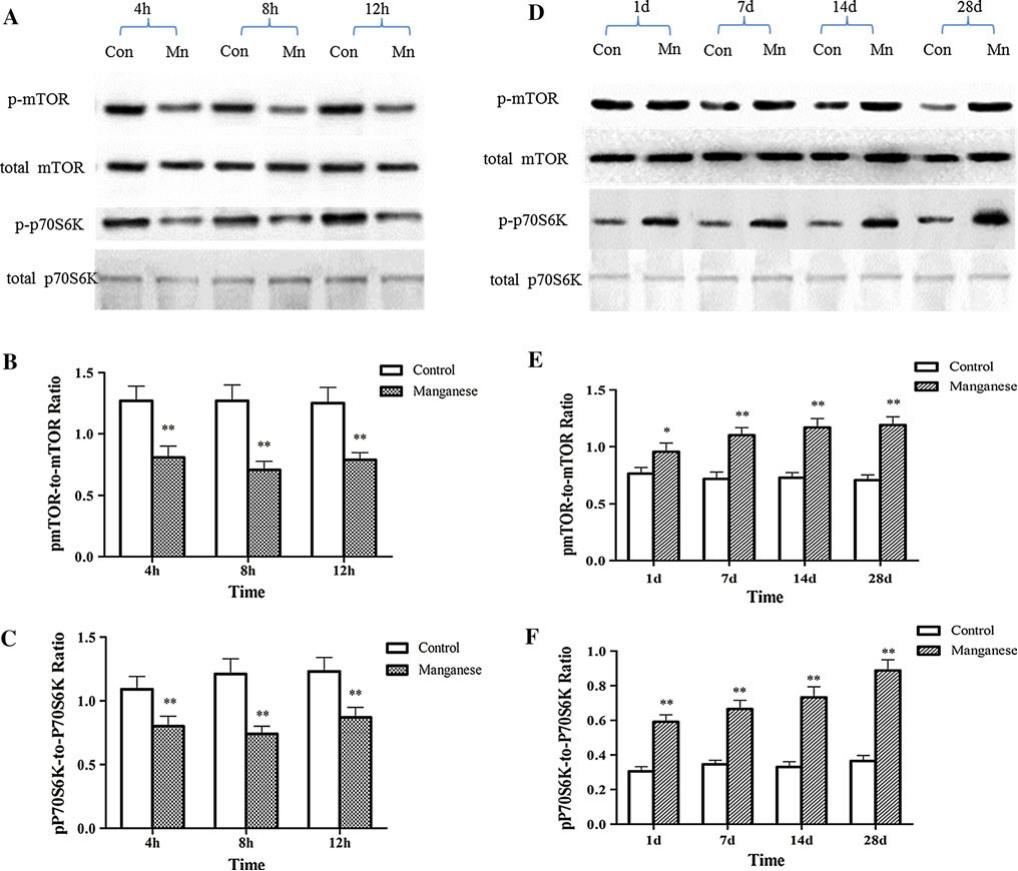
The effect of Mn on mTOR/p70S6K pathway. Short- (4, 8, and 12 h) and long-term (1, 7, 14, and 28 days) after Mn injection, Western blotting were conducted to evaluate autophagy-related signaling pathway mTOR/p70S6K. **a**, **d** Effect of Mn on mTOR/p70S6K phosphorylation after the short- and long-term exposure. **b**, **c**, **e**, and **f** Densitometry analysis of mTOR/p70S6K phosphorylation relative to total mTOR/p70S6K levels was performed in three independent experiments (mean ± SD; one-way ANOVA with Newman–Keuls post hoc analysis, *p < 0.05 and **p < 0.01)

The mammalian target of rapamycin (mTOR) and p70 ribosomal protein S6 kinase (p70S6K) are known to regulate antophagy, and the phosphorylation of these two kinases can suppress autophagy. In order to detect if this signaling pathway is related to the change of autophagy level caused by manganese exposure. The activation of mTOR and p70S6K was measured by western blotting. As shown in Fig. 5a, at short-time points post intrastriatal Mn injection (4, 8, and 12 h), the levels of phosphorylated mTOR and p70S6K were significantly reduced, implicating mTOR and p70S6K inhibition in the activation of autophagy. However, with the extension of manganese exposure time, mTOR/p70S6K pathway was significantly and time-dependently (1, 7, 14, and 28 days) activated in Mn-injected animals versus controls (Fig. 5d), which was consistent with the change of autophagy level.

### The Inhibition of Autophagy Promoted the Short-Term Neurotoxic Effect of Mn

**Fig. 6:**
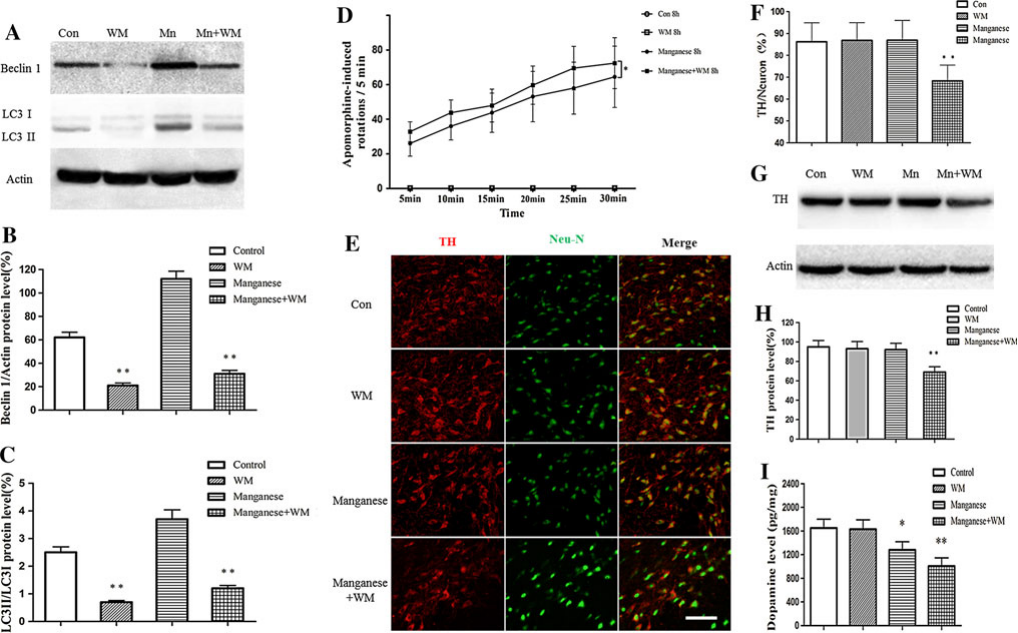
The inhibition of autophagy promotes the short-term effect of Mn on nigral DAergic neurons and apomorphine-induced rotations. After the pretreatment of wormannin in Mn-exposed rats, immunofluorescence and western blotting were conducted to measure TH-immunoreactivity and the expression of TH protein, and the apomorphine-induced rotations were also observed to measure manganese-induced Daergic neurotoxicity. **a** Effect of wortmannin on autophagy. **b**, **c** Densitometry analysis of Beclin 1 protein levels and LC3 II levels relative to LC3 I (mean ± SD; one-way ANOVA with Newman-Keuls post hoc analysis, *p < 0.05 and **p < 0.01). **d** Quantitative analysis of the effect of autophagy inhibition before Mn exposure on the apomorphine-induced rotations. **e** Effect of autophagy inhibition on manganese-induced TH-immunoreactivity change. **f** Quantitative analysis of the effect of autophagy inhibition on manganese-induced loss of DAergic neurons. *p < 0.05, **p < 0.01 compared with control groups, *scale bar* indicates 200 μm. **g** The inhibition of autophagy by wortmannin promotes the short-term effect of manganese on TH expression. **h** Densitometry analysis of TH levels relative to β-actin was performed after three independent experiments. **i** Effect of autophagy inhibition on manganese-induced dopamine levels change (mean ± SD; one-way ANOVA with Newman–Keuls post hoc analysis, *p < 0.05 and **p < 0.01)

To further evaluate the effect of autophagy on Mn-induced nigral DAergic neuron damage, we pretreated rats with wotmannin, an inhibitor of phosphatidylinositol 3′-kinase (PI3K III)/protein kinase B (Akt) and frequently used as an autophagy inhibitor, before Mn exposure. Then we conducted a behavioral observation. Although apomorphine-induced rotation increased compared with that of the control, with the injection of wortmannin, an autophagy inhibitor, more serious apomorphine-induced rotation was observed. Immunostaining results with primary antibodies against TH and Neu-N showed that the number of TH-immunoreactive and TH/Neu positive cells were indistinguishable between Mn-exposed rats (8 h post injection) and controls (Fig. 6e). Consistent with these results, the protein expression of TH was unaltered 8 h post Mn injection. However, in rats pretreated with wortmannin, Mn led to a significant decrease in the ratio of TH/Neu (Fig. 6e) and the TH protein level (Fig. 6g) at 8 h post-exposure. In addition, wortmannin significantly decreased dopamine and DA receptor levels and promoted Mn-induced behavioral abnormality, as shown by increased apomorphine-induced rotations (Fig. 6d, i). The above results indicate that the activation of autophagy may play an important role in protecting DAergic neurons against Mn-induced toxicity at short-time points post intrastriatal Mn injection.

## Discussion

As a ubiquitous constituent of the environment, Mn is an essential metal for animal and human health (Hearn et al. 2003). However, when excessive exposure to Mn occurs, it may cause a neurodegenerative disorder known as ‘‘manganism’’. The disorder is characterized as a Parkinson’s-like disease (BS and WJ 2003; Takeda 2003) and has been observed in miners, ferroalloy, and battery manufacture workers, as well as automotive repair workers (Racette et al. 2011). Work by others and us has established the propensity of Mn to induce DAergic neurodegeneration (Liu et al. 2009; Zhang et al. 2009). However, the mechanisms underlying this effect have yet to be fully clarified. Herein, we examined whether a single unilateral injection of MnCl_2_ into the caudate-putamen led to dysregulated autophagy and DAergic neurodegeneration. Since Mn was previously reported to be axonally transported in the GABAergic and DAergic nigrostriatal pathways (Liu et al. 2009; Zhao et al. 2009), the caudate putamen has been targeted as the primary site of Mn injection and the ensuing damage to the nigrostriatal DAergic pathway.

Autophagy is a general term for pathways by which cytoplasmic materials, including soluble macromolecules and organelles, are delivered to lysosomes for degradation (Levine et al. 2011). Autophagy is induced by a variety of stress stimuli, including nutrient and energy stress, ER stress, pathogen-associated molecular patterns and stress-associated molecular patterns, hypoxia, redox changes, and mitochondrial damages (Kroemer et al. 2010). The importance of autophagy to neurons has only been demonstrated recently (Mizushima et al. 2008). For example, mice lacking brain expression of Atg5 or Atg7 rapidly develop neurodegenerative phenotypes (Hara et al. 2006; Komatsu et al. 2006), indicating a neuroprotective role of autophagy. In addition, it has been reported that in early Alzheimer’s disease, the expression of the autophagy-related protein Beclin 1 is significantly reduced (Pickford et al. 2008). However, contradictory results have also been reported, as evidenced by the accumulation of autophagosomes in brain tissues of patients with Alzheimer’s disease (Nixon et al. 2005) and in a model of Huntington’s disease (Kegel et al. 2000). Therefore, in this study, we investigated the effect of Mn on autophagy and the role of its dysregulation in Mn-induced DAergic neuronal injury.

Our results showed that different time points (4 h, 8 h, 12 h, 1, 7, 14, and 28 days) after a single intrastriatal injection of Mn in rats, apomorphine-induced rotations were significantly increased. In order to expound the behavior change after injection of Mn in rats, we tested the dopamine levels, DA receptor and TH protein expression after Mn injection. It had been proved that dopamine levels decreased in the basal ganglia of monkey intoxicated with manganese and in rat striatum directly injected with manganese(Bird et al. 1984; Lista et al. 1986), our experimental results also proved that the dopamine levels and DA receptor protein expression were significantly decreased (Figs. 1, 2). Furthermore, TH-immunoreactivity of DAergic neurons and TH protein expression were significantly inhibited by Mn 1, 7, 14, and 28 days after the injection (Fig. 3). These results suggested that Mn exposure-affected dopamine levels and DA receptor expression, and leaded to the change of behaviors. As the extension of Mn exposure, Mn-caused irreversible damage to the DAergic neurons, reduced dopamine levels and D_1_ dopamine receptor expression, consequently influenced motion behaviors. These changes were accompanied by decreased number of autophagic vacuoles and increased number of dysfunctional lysosomes as noted by TEM (Fig. 4). Beclin1, a 60-kDa coiled-coil protein, is a Bcl-2-interacting cellular protein (LIANG et al. 1998) and a central regulator of autophagy nucleation and induction (Kroemer et al. 2010). Under stress condition, Beclin1 is activated by PI3KIII, resulting in the formation of autophagic vacuoles. Upon induction of autophagy, the microtubule-associated protein 1 LC3 is recruited to the membrane and lipidated with phosphatidylethanolamine to form LC3-II, a fast migrating form distinct from a cytosolic LC3-I form (Kabeya et al. 2000). LC3-II is located at specific sites in the inner and outer membranes of autophagasomes during early periods of autophagy and is essential for autophagosome formation (Kabeya et al. 2000). Thus, the ratio of LC3 II to LC3 I is used as a marker for autophagy induction. Our results showed that short-Mn exposure compensatorily increased Beclin1 protein expression and decreased ratio of LC3 II to LC3 I, suggesting the inhibited autophagy after long-term Mn exposure. The data demonstrated that Mn-induced DAergic neurodegeneration was associated with the inhibition of autophagy (Fig. 4).

It is well established that mTOR kinase, which integrates upstream signaling pathways, serves as a key signaling molecule in the suppression of autophagy (Mathew et al. 2007; Jung et al. 2010; Neufeld 2010). It has also been reported that the inhibition of p70S6K, a downstream target of mTOR signal, is involved in the enhancement of autophagy (Saiki et al. 2011). In order to test whether the inhibition of autophagy is dependent on the activation of mTOR and p70S6K signaling, phosphorylation levels of these kinases were evaluated, pursuant to Mn injection. As expected, 1–28 days after Mn exposure, both mTOR and p70s6K were significantly activated (Fig. 5d). Since the activated mTOR and p70s6K signaling are involved in the inhibition of autophagy (Saiki et al. 2011), it likely the activation of mTOR and p70S6K signaling, at least partly, was responsible for Mn-induced inhibition of autophagy.

Autophagy always represents an adaptative process, affording rapid protection against insults and stress. Accordingly, in this study, the early effects (<12 h) of Mn on autophagy were also examined. 4–12 h after Mn injection, autophagy was significantly activated, as evidenced by increased number of autophagosome and increased expression of Beclin1 and LC3 II (Fig. 4). These effects occurred concomitantly with the inhibition of mTOR/p70S6K signaling, the latter was likely responsible for the initiation of autophagy. Other reports have indicated that autophagosome formation requires PI3K activity and that PI3K inhibitors, such as wortmannin, LY294002, and 3-MA are typical autophagy inhibitors (Blommaart et al. 1997, Itakura et al. 2008, Matsunaga et al. 2009). TH-immunoreactivity and TH protein expression were not altered by intrastriatal Mn injection after short-term exposure (4, 8, and 12 h), but Mn-induced the down-regulation of dopamine level and D_1_ dopamine receptor expression, co-inhibition of the class III PI3K/Beclin 1 by pre-treatment with wortmannin significantly inhibited TH-immunoreactivity and TH/Neu ratio in the SNpc of the rats exposed to Mn. In addition, pretreatment with wortmannin significantly reduced the dopamine levels and increased apomorphine-induced rotations in rats exposed to Mn, suggesting that in the early periods post Mn exposure, activated autophagy (Fig. 6) may be compensatorily against neurotoxicities associated with Mn (Fig. 5).

The exact role of dysregulated autophagy in various diseases has yet to be delineated. Notably, as of date, with the exception of this study, no others have assessed the role of autophagy in Mn-induced neurotoxicity. Herein, we demonstrated that a single injection of Mn in rats led to DAergic neurodegeneration and abnormal apomorphine-induced behaviors 1–28 days after injection. Concomitant with these effects, Mn led to the inhibition of autophagy. In contrast, at earlier time-points post injection (4–12 h), autophagy was activated by Mn, likely reflecting transient protection against Mn-induced DAergic neurodegeneration. In conclusion, these data shed novel information on the role of dysregulation of autophagy in Mn-induced neurodegeneration, which may be pharmacologically target to ameliorate the aberrant effects of this metal.

## Abbreviations

CNS: Central nervous system
CPu: Caudate–putamen complex
LC3: Microtubule-associated protein 1 light chain 3
Mn: Manganese
mTOR: Mammalian target of rapamycin
p70s6k: p70 Ribosomal protein S6 kinase
PD: Parkinson’s disease
SN: Substantia nigra
TH: Tyrosine hydroxylase
